# Evaluation of Cross-Talk and Alleviate Potential of Cytotoxic Factors Induced by Deoxynivalenol in IPEC-J2 Cells Interference with Curcumin

**DOI:** 10.3390/ijms25136984

**Published:** 2024-06-26

**Authors:** Qiyuan Wang, Aike Li, Hao Yu, Chuanqi Wang, Ting Wang, Jing Zhang

**Affiliations:** 1 Academy of National Food and Strategic Reserves Administration, Beijing 100037, China; qiyuanw22@mails.jlu.edu.cn (Q.W.); lak@ags.ac.cn (A.L.); 2 College of Animal Sciences, Jilin University, Changchun 130062, China; yu_hao@jlu.edu.cn (H.Y.); wangchuanqi@jlu.edu.cn (C.W.); wangting23@mails.jlu.edu.cn (T.W.)

**Keywords:** deoxynivalenol, curcumin, cross-talk, cytotoxic factors, IPEC-J2 cells

## Abstract

Deoxynivalenol (DON) is a mycotoxin produced by *Fusarium graminearum*, and curcumin (CUR) is a natural polyphenolic compound found in turmeric. However, the combined treatment of CUR and DON to explore the mitigating effect of CUR on DON and their combined mechanism of action is not clear. Therefore, in this study, we established four treatment groups (CON, CUR, DON and CUR + DON) to investigate their mechanism in the porcine intestinal epithelial cells (IPEC-J2). In addition, the cross-talk and alleviating potential of CUR interfering with DON-induced cytotoxic factors were evaluated by in vitro experiments; the results showed that CUR could effectively inhibit DON-exposed activated TNF-α/NF-κB pathway, attenuate DON-induced apoptosis, and alleviate DON-induced endoplasmic reticulum stress and oxidative stress through PERK/CHOP pathways, which were verified at both mRNA and protein levels. In conclusion, these promising findings may contribute to the future use of CUR as a novel feed additive to protect livestock from the harmful effects of DON.

## 1. Introduction

**Figure 1:**
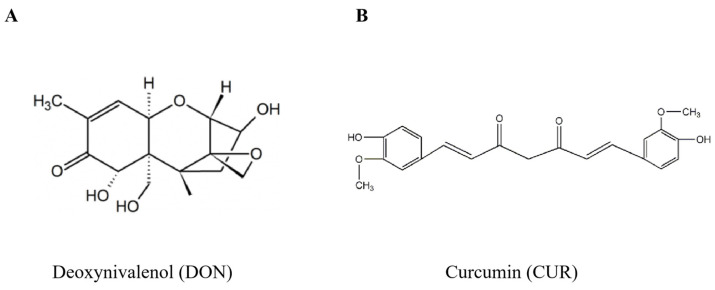
(**A**) Chemical Structures of Deoxynivalenol. (**B**) Chemical Structures of Curcumin.

Mycotoxins are secondary metabolites produced by fungi under appropriate temperature, moisture and nutritional conditions, which at certain doses have adverse effects on humans and animals, including carcinogenicity, immunotoxicity, hepatotoxicity and gastrointestinal toxicity [1]. It is estimated that there are more than 400 mycotoxins worldwide, and common mycotoxins include deoxynivalenol (DON), aflatoxin, zearalenone, fumonisin and ochratoxin [2]. DON, among other mycotoxins, has attracted attention because of its widespread distribution and high toxicological potential [3]. DON is a type B trichothecene mycotoxin produced by the catabolism of *Fusarium graminearum* with a 12,13-epoxy-trichothec-9-ene skeleton (Figure 1A) and is widely distributed in corn, wheat, rice, oats, etc [4]. The double bond between the C9 and C10 sites and the 12,13-epoxide ring are key structural elements in the toxicity of DON and other trichothecenes [5]. DON has been shown to cause vomiting, food refusal, diarrhoea, growth retardation, gastrointestinal bleeding, immune system disorders and neurological disease in animals. Pigs are particularly susceptible to DON [6]. The predominant target organ for DON action is the intestine [7], which is the primary site for digestion and absorption of nutrients and is the first barrier to the invasion of pathogenic microorganisms and external toxins when exposed to high concentrations of mycotoxins through the ingestion of contaminated feed [8]. A large body of evidence suggests that when DON enters the intestinal epithelial cells by passive diffusion after feed ingestion, it inhibits the protein synthesis of the intestinal epithelial cells, disrupts the structure of tight junction proteins, damages the integrity of the intestinal barrier [9,10,11], and increase the permeability of the intestinal epithelium, which would reduce nutrient absorption and transfer efficiency, and ultimately reduce production performance.

In recent years, there has been increasing interest in epidemiological studies of the relationship between mycotoxin exposure and cancer risk, with some reports of associations between mycotoxin exposure and primary liver, breast and cervical cancer [12]. Experiments have found that oral exposure of mice to DON for 8 days leads to increased intensity of enteritis and development of colorectal cancer, demonstrating the cancer-promoting effects of DON [13]. A number of molecular mechanisms related to DON toxicity have been identified, such as DON, which could activate TNF-α and further activate the NF-κB signalling pathway; it could also induce oxidative stress and cause mitochondrial damage, leading to the release and accumulation of ROS [14]. Meanwhile, apoptosis, MAPK, JAK/STAT, endoplasmic reticulum stress (ERS) and other pathways are also involved in the DON toxicity response [15,16,17]. It is well known that TNF-α is involved in the maintenance and homeostasis of the immune system and is closely associated with cancer development [18], while NF-κB is the major downstream target of TNF-α signalling [19], which highlights the importance of the TNF/NF-κB signalling pathway in immune activity. Several studies have found that DON treatment will activate such pathway; for example, when IPEC-J2 cells were treated with DON, it was found that the relative mRNA expression of NF-κB, IL-1β and IL-6 increased with increasing DON concentration, and the expression of NF-κB protein gradually enhanced [20]. While DON-contaminated diets were fed to weaned piglets, it was shown that DON upregulated serum levels of TNF-α and increased TNF-α protein expression and mRNA expression of NF-κB [21]. Likewise, the effect of DON treatment on apoptosis is also significant. It was found that 30 μM DON induced apoptosis in prostate epithelial cells by modulating the PI3K/Akt signalling pathway [15]. DON also induced apoptosis in porcine intestinal epithelial cells via the FOXO3a pathway and decreased the mRNA expression of Bcl-6, caspase-3 and caspase-8 [22]. Regarding DON-induced ERS, it has been reported that DON could trigger ERS by activating the PERK/CHOP signalling pathway under vitro conditions, resulting in misfolded or unfolded protein response (UPR) in it [23,24]. This will result in the accumulation of misfolded and unfolded proteins in the space of the endoplasmic reticulum along with the disturbance of Ca^2+^ imbalance, a loss of function, and ultimately, the disruption of intracellular redox homeostasis, bringing about the accumulation of reactive oxygen species (ROS) [25], leading to cell damage or apoptosis, which is a kind of cellular protective stress response [26,27]. The above expression indicates that in a realistic environment, it is clear that DON exposure has caused serious negative impacts, so it is imperative to find an effective and safe compound to mitigate the damage it causes.

Curcumin (CUR) is an abundant compound in turmeric plants. It has been attracted by its excellent antioxidant, anticancer, and anti-inflammatory properties [28]. Chemically, it is identified as 1,6-heptadiene-3,5-dione-1,7-bis (4-hydroxy-3-methoxypheny l)-(1E,6E) (Figure 1B) [29]. The activity and bioavailability of CUR depend mainly on three reaction sites: hydrogen atom transfer, Michael receptor and metal chelator [30]. The o-methoxyphenol moiety and methylidene hydrogen are the main components of CUR’s antioxidant activity, and CUR interacts with a wide range of biomolecules through non-covalent and covalent binding. The α, β-unsaturated β-diketone molecule covalently interacts with protein thiols through the Michael reaction. The β-diketone moiety forms a chelate with the transition metals, thereby reducing metal-induced toxicity. Meanwhile, the hydroxyphenyl molecule in CUR has been shown to be the key to its anti-inflammatory activity. In recent decades, although some derivatives based on the partial structural modification in the aromatic and the diketo moiety of curcumin have shown promising activity, none of them is as effective as curcumin in terms of multiple effects [31]. There is a body of evidence suggesting that CUR may exert its immunoregulatory capacity by interacting with several immune mediators, mainly associated with some inflammatory pathways, such as the TNF/NF-κB pathways [32,33,34]. The main mechanism of NF-κB activation is the phosphorylation of the multi-subunit IkBa kinase complex to IκBα, and upon activation, NF-κB is translocated to the nucleus to bind to specific shared DNA sequences, thereby enabling transcription to function [35]. At the same time, CUR can suppress NF-κB activity by inhibiting the phosphorylation of IκBa and blocking the nuclear translocation of the NF-κB p65 subunit [36]. In addition, just as TNF-α activates NF-κB, which then expresses inflammatory genes, CUR may block the transcription of TNF-α, thereby blocking the expression of inflammatory genes [37]. CUR’s effect on apoptosis is also remarkable. On the one hand, it is able to induce apoptosis in a variety of tumour cell lines, activate intracellular redox reactions and induce ROS production, thereby inhibiting tumour cell proliferation. For example, CUR can downregulate Notch signalling and induce apoptosis and G0/G1 blockade in DU-145 prostate cancer cells [38]. It induces ROS activation of the KEAP1/NRF2/ARE pathway and induces the expression of miR-34a and miR-34b/c, leading to apoptosis and senescence in colon cancer cells [39]. On the other hand, CUR is able to attenuate apoptosis induced by radiation or toxicity in normal tissues and to exert a protective effect, which was found to rescue the LPS-induced decrease in MAC-T cell viability and cellular damage, which may be related to the inactivation of NF-κB and Bcl-2 pathways [40]. It is interesting to note that there may be a bidirectional relationship between ERS and the NF-κB pathway, with ERS activating the NF-κB pathway and NF-κB regulating the UPR, which interact to form a feedback loop [41]. At the same time, CUR has been shown to exert a protective effect by modulating ERS and inflammation through a mechanism that inhibits the UPR and reduces the expression of inflammatory cytokines and chemokines [42]. It is reported that CUR can inhibit the expression of markers associated with ERS and NF-κB pathways in LPS-induced chronic inflammatory mice [43]. It was also found that feeding CUR to piglets under diquat-induced oxidative stress revealed that CUR improved mitochondrial function and ultrastructure, alleviated ERS and inhibited apoptosis induced by diquat [44]. Unfortunately, CUR has low bioavailability, which may be related to its low serum levels, limited tissue distribution, apparently rapid metabolism and short half-life [45]. It was found that oral administration of 0.5 g of CUR in rats resulted in a maximum residue level of only 60 ng/mL [46], and as in humans, administration of CUR at a dose of 2 g/kg resulted in undetectable or very low (0.006 ± 0.005 μg/mL) serum levels after 1 h [47]. However, current novel delivery strategies, including nanoparticles, liposomes and defined phospholipid complexes, offer great promise for improving the absorption and bioavailability of CUR.

In the present study, we suppose that DON has activated cytotoxic factors such as TNF-α, NF-κB, apoptosis and ERS, whereas CUR can modulate the intracellular redox level, attenuate DON-induced inflammatory response, apoptosis and ERS, and gradually alleviate cellular damage, thus mitigating the hazards of DON-contaminated diets in actual production. Therefore, this experiment evaluated the cross-talk of CUR interfering with DON-induced cytotoxic factors using IPEC-J2 cells as a model and elucidated the specific regulatory mechanism of CUR to alleviate DON-induced apoptosis and other damages.

## 2. Results

### 2.1. Cross-Talk between CUR Targets with DON-Induced Cytotoxicity Factors

**Figure 2:**
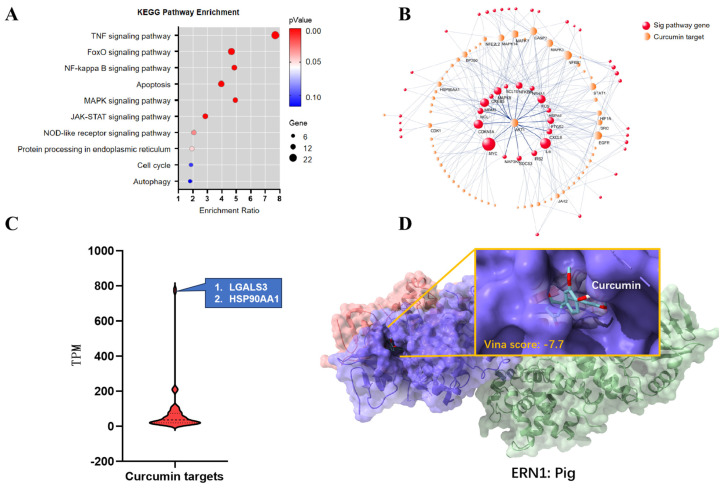
Cross−talk between CUR Targets with DON−induced Cytotoxicity Factors. (**A**) Top 10 enriched KEGG pathways for up−regulated. (**B**) Protein−protein interaction (PPI) network of curcumin target and genes on the top 8 up−regulated pathway significantly (*p* < 0.05) enriched KEGG pathways (only the nodes with connectivity greater than five are labelled on the network). (**C**) Violin plot for TPM distribution of 66 curcumin targets. (**D**) Molecular docking (Autodock result of the complex structures) showing the interaction of curcumin and porcine ERN1.

To assess the effectiveness of CUR in attenuating the cytotoxicity of DON, we first identified the top 10 up-regulated pathways (54 genes) from the public transcriptome dataset (PRJNA578240) and, at the same time, predicted 444 CUR targets from three public servers (PharmMapper, SuperPred and GalaxySagittarius). Based on Stringdb, we finally screened 66 CUR targets with high mRNA expression levels interacting with 51 genes enriched in the eight up-regulating pathways (confidence level > 0.7), and the PPI network indicated that all 17 hub genes in the eight pathways were all cooperate with AKT1 (hub target) and secondary targets to interact with secondary pathway genes (Figure 2A,B). Next, we further checked the mRNA expression of CUR targets and found that both LGALS3 and HSP90AA1 are extremely highly expressed targets with TPM values more than 70-fold of the overall mean TPM value, and they are also the novel activators of the TNF-α and NF-κB signalling pathways (Figure 2C). We also found that ERN1 is not only a target of CUR but is also a significant up-regulated gene in the endoplasmic reticulum, and molecular docking results showed that CUR was able to bind into the same pocket of GSK2850163A, an inhibitor of human ERN1 in the porcine ERN1 structure, as shown in Figure 2D. In addition, other targets, such as Caspase3, NF-κB and MAPKs, all reveal that CUR can block the cross-talk between targets and pathway genes at the protein level, thus alleviating the cytotoxicity caused by the activation of TNF, NF-κB, apoptosis and endoplasmic reticulum stress pathways.

### 2.2. Evaluation of the Efficacy of CUR in Alleviating Cytotoxicity of IPEC-J2 Induced by DON

**Figure 3:**
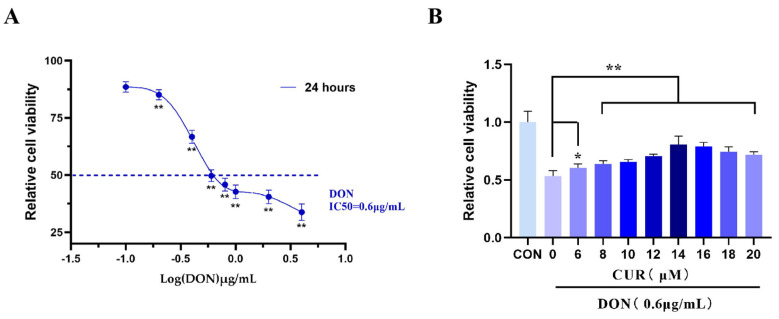
Evaluation of the Efficacy of CUR in Alleviating Cytotoxicity of IPEC−J2 Induced by DON. (**A**) The relative cell viability of IPEC−J2 cells exposed to DON for 24 h. (**B**) The relative cell viability of IPEC−J2 cells exposed to DON (0.6 μg/mL) combined with CUR for 24 h. * *p* < 0.05, ** *p* < 0.01, compared to 0.6 μg/mL DON + 0 μM CUR.

To evaluate the protective effect of CUR on IPEC-J2 cells exposed to DON, we determined the viability of cells pretreated with CUR, DON and CUR + DON for 24 h using the CCK-8 assay. When IPEC-J2 cells were first treated with different concentrations of DON (0.1, 0.2, 0.4, 0.6, 0.8, 1.2 and 4 μg/mL), it was observed that DON was able to induce IPEC-J2 cell death in a dose-dependent manner (Figure 3A), and the IC_50_ value was 0.6 μg/mL. Subsequently, different concentrations (6, 8, 10, 12, 14, 16, 18, and 20 μM) of CUR were used to treat IPEC-J2 cells exposed to DON (IC_50_), and 14 μM CUR was found to be the most effective in alleviating cell viability (Figure 3B), therefore 14 μM CUR and 0.6 μg/mL DON were selected for the following experiments to treat cells for 24 h.

### 2.3. Verification of CUR on the Inhibition of TNF-α/NF-κB Signaling Pathways Induced by DON

**Figure 4:**
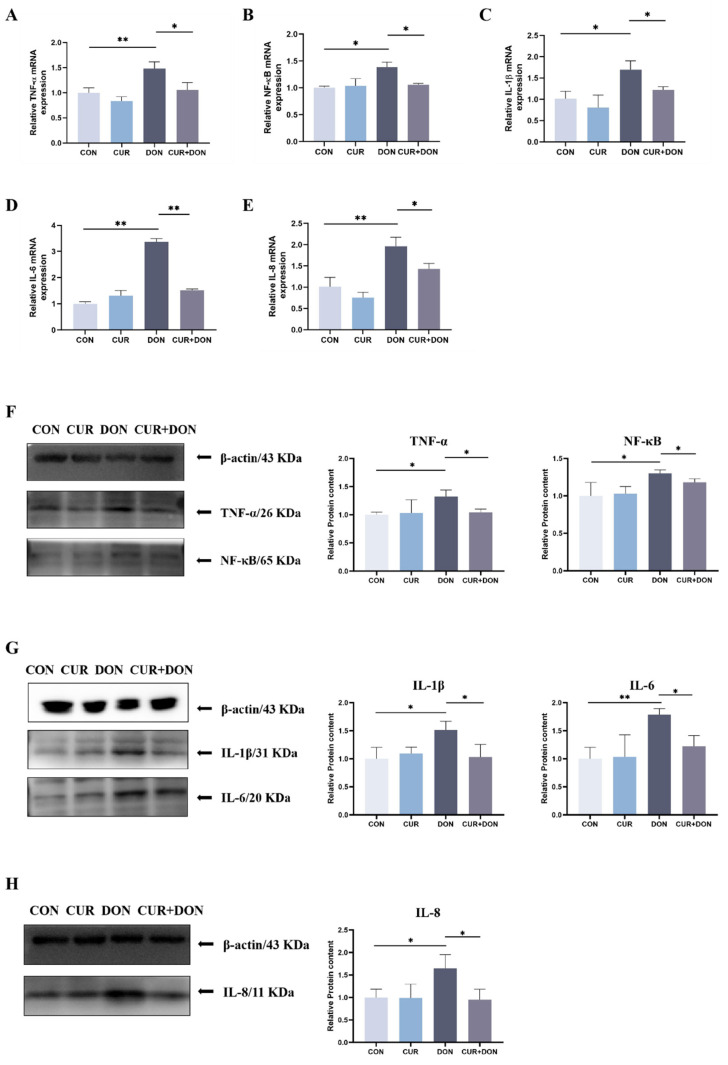
Verification of CUR on the Inhibition of TNF-α/NF-κB Signaling Pathways Induced by DON (**A**) Relative TNF-α mRNA level. (**B**) Relative NF-κB mRNA level. (**C**) Relative IL-1β mRNA level. (**D**) Relative IL-6 mRNA level. (**E**) Relative IL-8 mRNA level. (**F**) Relative protein levels of TNF-α and NF-κB. (**G**) Relative protein levels of IL-1β and IL-6. (**H**) Relative protein levels of IL-8. * *p* < 0.05, ** *p* < 0.01, compared to control (CON) and deoxynivalenol (DON).

To validate that CUR inhibition of DON triggers TNF-α and NF-κB activity in IPEC-J2 cells, we validated five markers at the mRNA and protein levels. These data showed that the mRNA and protein levels of TNF-α, NF-κB, IL-6, IL-8 and IL-1β were greatly increased by the DON group compared with the CON group. Compared with the DON-treated group, the mRNA and protein levels of TNF-α, NF-κB, IL-6, IL-8 and IL-1β were decreased in the CUR + DON group (Figure 4A–H), suggesting that DON induced the expression of inflammation-associated cytokines in IPEC-J2 cells, while CUR treatment could attenuate the DON-induced deleterious effects.

### 2.4. CUR Treatment Rescues the Apoptosis of IPEC-J2 Cells Induced by DON

**Figure 5:**
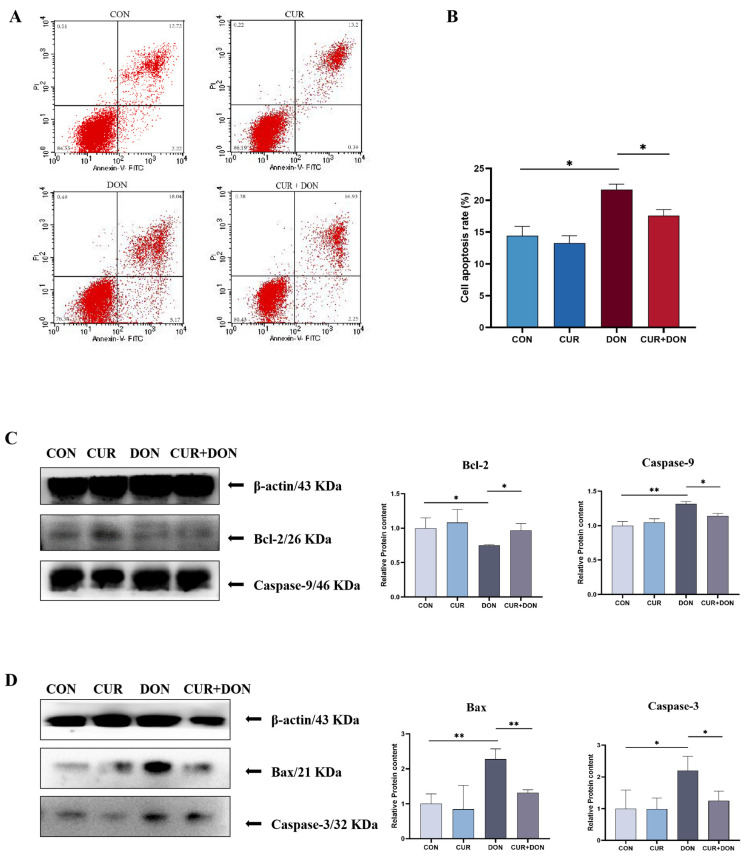
CUR Treatment Rescues the Apoptosis of IPEC-J2 Cells Induced by DON. (**A**) Cell death was assessed by annexin V/PI staining. (**B**) Cell apoptosis rate. (**C**) Relative protein levels of Caspase-9 and Bcl-2. (**D**) Relative protein levels of Bax and Caspase-3. * *p* < 0.05, ** *p* < 0.01, compared to control (CON) and deoxynivalenol (DON).

To examine the influence of DON and CUR on apoptosis in IPEC-J2 cells, we detected the apoptosis rate in IPEC-J2 cells using flow cytometry, and it was found that the apoptosis rate was dramatically increased after 24 h of DON treatment of the cells, in contrast to CUR, which could substantially rescue the DON-induced apoptosis (Figure 5A,B). Furthermore, the effect of CUR and DON on apoptosis-associated proteins was assessed by western blot. As shown in Figure 5C,D, DON treatment increased the protein levels of caspase-3, caspase-9 and Bax and markedly decreased Bcl-2 protein in IPEC-J2 cells compared to the CON group. The CUR + DON group was found to be able to resist such effects more than the DON group. These studies all showed that DON treatment had a post-apoptotic effect on IPEC-J2 cells but that CUR had an effect of arresting DON-induced apoptosis.

### 2.5. CUR Treatment Reduces DON-Induced ERS through PERK/CHOP Pathways

**Figure 6:**
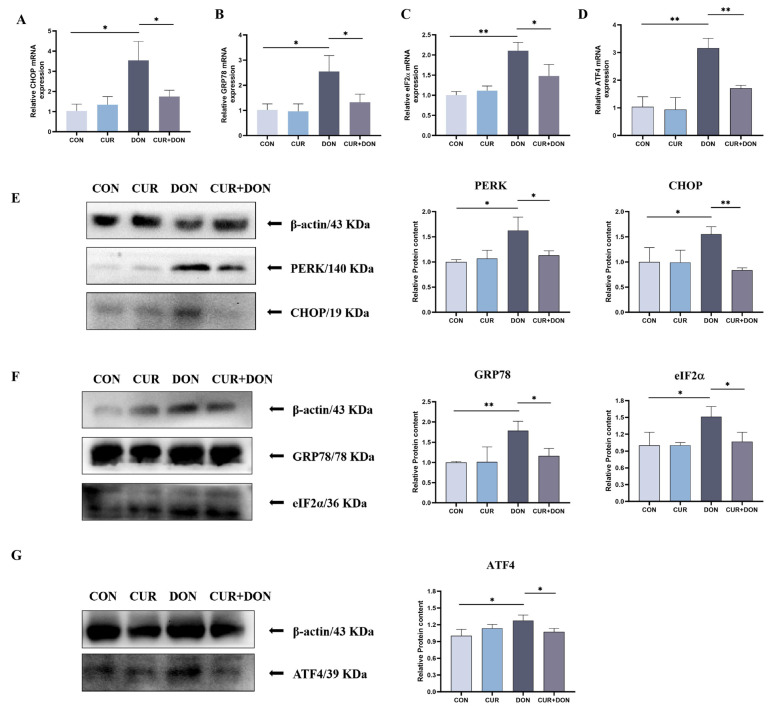
CUR Treatment Reduces DON-Induced ERS through PERK/CHOP Pathways. (**A**) Relative CHOP mRNA level. (**B**) Relative GRP78 mRNA level. (**C**) Relative eIF2α mRNA level. (**D**) Relative ATF4 mRNA level. (**E**) Relative protein levels of PERK and CHOP. (**F**) Relative protein levels of GRP78 and eIF2α. (**G**) Relative protein levels of ATF4. * *p* < 0.05, ** *p* < 0.01, compared to control (CON) and deoxynivalenol (DON).

Based on the hints of result 1, it is possible that ERS is a major pathway responsible for DON-induced oxidative stress and involves more than two targets with high binding capacity for CUR. To determine the effects of CUR and DON on ERS in IPEC-J2 cells, we measured the expression of relevant mRNAs and proteins in the PERK/CHOP pathways. The expression of relevant mRNAs by RT-qPCR, as shown in Figure 6A–D, DON treatment individually increased the gene expression of GRP78, eIF2α, ATF4 and CHOP in cells compared to the CON group, and the combined treatment of CUR + DON resulted in a significant decrease in the expression levels of these four genes compared to the DON group. Further confirmation by western blot, as shown in Figure 6E–G, indicated that the protein levels of GRP78, PERK, eIF2α, ATF4 and CHOP in the cells were substantially increased by DON treatment. However, when CUR and DON were combined, the abundance of these five proteins was downregulated greatly compared to the DON group. These results demonstrated that DON was able to activate the PERK/CHOP signalling pathways to induce ERS in cells, while CUR could effectively reduce the DON-induced adverse effects.

### 2.6. Cytological Validation of CUR to Attenuate DON-Induced Oxidative Stress in IPEC-J2 Cells

**Figure 7:**
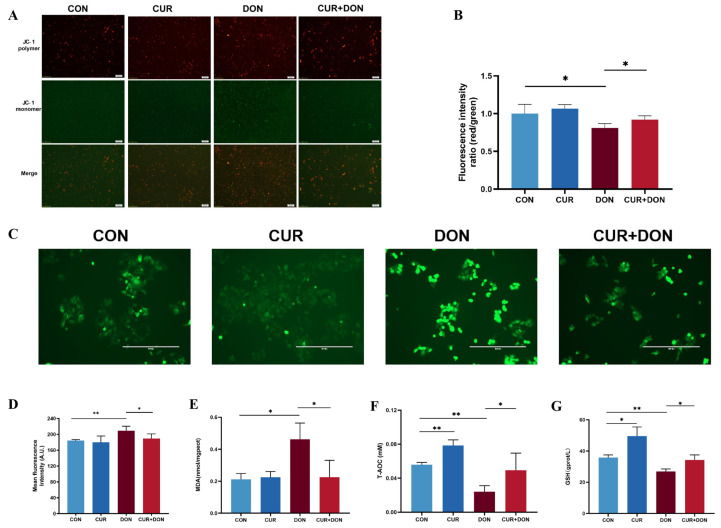
Cytological Validation of CUR to Attenuate DON-induced Oxidative Stress in IPEC-J2 Cells. (**A**) Inverted fluorescence microscopy of IPEC-J2 cells after JC-1 staining (scale bar represents 200 μm). (**B**) The different ratios (JC-1 polymer/JC-1 monomer) of JC-1 fluorescence for each group. (**C**) After incubation with DCFH-DA, cells were washed and examined by fluorescence microscopy (scale bar represents 400 μm). (**D**) Analysis based on green fluorescence intensity in (**C**). (**E**) MDA content in IPEC-J2 cells. (**F**) T-AOC activity in IPEC-J2 cells. (**G**) GSH activity in IPEC-J2 cells. * *p* < 0.05, ** *p* < 0.01, compared to control (CON) and deoxynivalenol (DON).

To further confirm that CUR attenuates oxidative stress in IPEC-J2 cells via the PERK/CHOP pathways, we first used the JC-1 fluorescent probe to determine the changes in MMP in IPEC-J2 cells under alternative treatments. It was shown that MMP was obviously decreased in the DON group compared with the control group, while the combination of CUR + DON allowed the cellular MMP level to be significantly increased compared with the DON group (Figure 7A,B), indicating that CUR could protect the cellular mitochondrial damage caused by DON by promoting energy metabolism. Next, the ROS results showed that compared with the CON group, DON treatment had significantly increased the level of ROS in IPEC-J2 cells. Furthermore, when cells were treated with DON + CUR, CUR significantly reduced DON-induced ROS accumulation compared to the DON group (Figure 7C,D). Finally, several antioxidant indicators were measured to explore the extent of DON-induced cellular oxidative stress and the alleviation by CUR [14,48]. The results showed that compared to the CON group, DON treatment significantly increased the levels of MDA (Figure 7E) and decreased the activities of T-AOC (Figure 7F) and GSH (Figure 7G) in IPEC-J2 cells. Furthermore, when cells were treated with DON + CUR, CUR greatly reduced DON-induced MDA levels and significantly increased T-AOC and GSH compared to the DON group. These data indicated that DON induced oxidative stress in IPEC-J2 cells, whereas CUR inhibited the oxidative stimulation of DON-exposed cells.

## 3. Discussion

In this research, we used IPEC-J2 cells as a research model to imitate the intestinal digestive environment in animals and stimulated the cells with appropriate concentrations of DON to comprehensively evaluate the effects of CUR in alleviating ERS and enhancing the cells’ anti-apoptotic and anti-inflammatory capabilities. On the one hand, DON is metabolised to produce toxic effects, and research on its metabolic pathways in animals and humans has revealed that DON is converted to the glucose-binding product, DON-3-O-glucoside (DON-3-Glc), which is involved in metabolic reactions. In humans, it is metabolised to free DON combined with DON-glucuronide (DON-GlcA); in animals, it can be converted to the C12,13-deepoxy metabolite, DOM-1, the conjugated products, DON-glucuronide, DON-sulfate and DOM-l-glucuronide [49]. CUR, on the other hand, has been shown to be safe in humans and animals as a non-genotoxic drug. Such safety has been demonstrated, for example, by the finding that humans exposed to oral CUR at a dose of 6 g/day for 4–7 weeks showed normal behaviour [50]. In animals, oral administration of CUR showed no major toxic effects, and five rats evaluated by CUR (single dose of 5000 mg/kg) by the oral route showed no adverse symptoms during a 14-day observation period [51]. In a mouse model of CUR pharmacotoxicity, the LD_50_ was greater than 21,500 mg/kg [52]. The metabolism of CUR in animals and humans has shown that, once absorbed, CUR binds to various tissue sites, such as sulphation and glucuronidation. Holder et al. showed that the major metabolites of CUR, when administered orally to rats, were glucuronides of tetrahydrocurcumin (THC) and hexahydrocurcumin (HHC), with minor metabolites being dihydroferulic acid and trace ferulic acid [53]. Pan et al. showed that curcumin glucuronoside, dihydrocurcumin glucuronoside, THC glucuronoside and tetrahydrocannabinol are the major metabolites of CUR in vivo [54]. Our results showed that CUR can effectively inhibit the reduction in cell viability induced by DON and alleviate the cell membrane damage caused by DON, suggesting that CUR not only promotes cell growth and metabolism but also attenuates the harmful effects of DON. CUR has been shown to be a potential adjunct to mitigate the damaging effects of mycotoxins such as DON on intestinal cells, as in the case of IPEC-J2. Indeed, extensive studies have been conducted on the in vitro protective properties of CUR, not only against DON but also against other mycotoxins. For example, CUR effectively reduced aflatoxin B1-induced toxicity in bovine fetal liver cell lines, resulting in an approximately 30% reduction in cell mortality [55]. In mesenchymal cells, CUR was able to attenuate zearalenone-induced oxidative stress and apoptosis via PI3K-AKT, Nrf2 and ER stress pathways [56]. CUR was able to protect against cytotoxicity induced by ochratoxin A, fumonisin B1 and DON in PK-15 cells, alleviate ochratoxin A-induced myotoxicity and promote the expression of relevant proteins and genes associated with ochratoxin A inhibition [57].

Tumour necrosis factor TNF-α is the key mediator and regulator of the mammalian immune response, controlling developmental regulation of the immune system, cell proliferation and signalling pathways, while TNF-induced NF-κB is its major biochemical function [58]. It has been shown that DON exposure induces the transcription of pro-inflammatory factors such as TNF-α, NF-κB, IL-1β, IL-6, and IL-8 [59,60], leading to an inflammatory response and involvement in a variety of cellular immune injuries and the target of this response is precisely the TNF/NF-κB pathway, considered to be the classical inflammation-associated pathway. The mechanism of the response is that NF-κB associates with its inhibitory protein IκBα and is located in the cytoplasm where they are inactive in normal cells; upon activation of the cell by TNF, IκBα is phosphorylated by the IKK complex containing IκB kinase (IKK), and the activated IKK complex in turn phosphorylates IκBα, leading to its degradation by the proteasome, which in turn activates NF-κB and transports it to the nucleus where it initiates or enhances transcription of the relevant genes [61]. In our research, the in vitro experiments showed that DON exposure induced an increase in the mRNA and protein expression of TNF-α, NF-κB and the downstream genes IL-1β, IL-6 and IL-8, demonstrating that DON induces cellular inflammatory responses through activation of the TNF/NF-κB pathway. While the NF-κB pathway has been implicated as one of the targets of CUR, a large body of evidence suggests that CUR can reduce the inflammatory response caused by external stimuli by inhibiting the activation of factors such as NF-κB, IL-8 and others [62,63]. The mechanisms lie in the fact that it inhibits the activation of IKK, the phosphorylation and degradation of IκBα, the nuclear translocation of NF-κB and the proteasomal function [64,65], thus inhibiting the activation of the NF-κB signalling pathway, which is consistent with our results showing that CUR effectively alleviated DON-induced inflammatory responses by inhibiting the activation of the TNF/NF-κB signalling pathway and exhibited potent anti-inflammatory properties.

Apoptosis is a type of programmed cell death that has two main mechanisms: extrinsic and intrinsic. Although both are essential, the intrinsic mechanism is more sensitive to the environment and DNA damage and involves mitochondria and mitochondrial proteins, and it is this mitochondrial apoptosis that is controlled by members of the BCL-2 gene family through the control of both pro-apoptotic and anti-apoptotic intracellular signals [66]. Of these, Bcl-2 is a representative anti-apoptotic protein, and Bax is an important pro-apoptotic protein. When cells are stimulated, the pro-apoptotic programme is activated, altering the expression of the Bcl-2 family of apoptosis-related proteins [67]. Bax changes conformation and accumulates on the outer mitochondrial membrane, causing a change in membrane permeability, leading to the release of cytochrome C from the mitochondria and activation of the caspase pathway [68]. Immediately following activation of the internal pathway caspase 9, the executioner caspase three is activated, leading to changes in nuclear and cytoplasmic morphology and, ultimately, cell death [69]. It has been previously reported that DON can induce an increase in the rate of apoptosis in hepatocytes, upregulate caspase-3 protein expression and Bax mRNA expression, activate the death receptor pathway and promote apoptosis [21], and we found that DON exposure upregulated the expression of Bax, caspase-9 and caspase-3 proteins, increased the rate of apoptosis and downregulated Bcl-2 protein, which is consistent with our findings. CUR, as a natural compound, has been shown in many studies to alleviate apoptosis induced by external stimuli and to attenuate cellular damage. For example, CUR alleviates neuronal apoptosis and neuroinflammation induced by cerebral haemorrhage by inhibiting the JAK1/STAT1 pathway and inhibits IL-1β-induced chondrocyte apoptosis by activating autophagy and inhibiting the NF-κB pathway. In our results, CUR treatment attenuated DON-induced apoptosis, which is consistent with previous findings and illustrates the potent anti-apoptotic property of CUR.

Oxidative stress has been defined as the deregulation between the production of ROS and intrinsic antioxidant defence mechanisms [70], with the intestinal epithelium being located in the interface between the organism and lumen, making it susceptible to oxidative damage evoked by oxidants [71]. It has previously been demonstrated that oxidant induction can contribute to the intensification of ROS production in the intestinal system, causing inadequate defence and promoting the establishment of intestinal pathologies [72]. Like the majority of cells, the principal endogenous components of the antioxidant defence mechanism which are responsible for ROS inactivation in intestinal cells are superoxide dismutase (SOD), catalase, glutathione peroxidase (GPx), nicotinamide adenine dinucleotide (NAD) and GSH etc. Some studies were reported earlier to have shown that DON exposure increased ROS production and inhibited antioxidant enzyme activities in IPEC-J2 cells [14], which is in agreement with our studies. In addition, high-calorie foods will release large amounts of free radicals, and the intake of DON for people or animals already in a state of high free radicals will aggravate the oxidation level of the body; DON releases free radicals, including ROS, which induces lipid peroxidation and alteration of the cellular antioxidant status and reduces the activity of antioxidant enzymes (such as GST, SOD and CAT) in vivo [73]. Some researchers have investigated the mechanism of interaction between ROS (more specifically, O atoms, OH radicals, H_2_O_2_ molecules and O_3_ molecules) and DON molecules at the atomic level using RMD simulations. The data showed that due to the high oxidative potential of ROS, the intact structure of DON is disrupted, and an H_2_O_2_ molecule breaks down into two OH radicals, while O_3_ molecules break down into an O_2_ molecule and an O atom, with the O atom extracting hydrogen (H) atoms from the DON molecule to form OH radicals that can be bonded to the C13 site. 12,13- The epoxide ring is disrupted by a ring-opening reaction, and the formation of aldehyde and ketone groups can also be observed during the H-abstraction reaction; in addition to ketone radicals formed on the epoxide ring, ketone radicals are formed at the C3-OH, C7-OH and C4-methylene sites [74].CUR is a protective antioxidant that can scavenge ROS and inhibit oxidative damage [75,76]. In vitro experiments with CUR demonstrated that it not only scavenges intracellular reactive oxygen species but also stimulates antioxidant response elements to restrain reactive oxygen species-induced oxidative stress [39]. The current results revealed that curcumin could reduce DON-induced accumulation of ROS and MDA and also increase the activities of antioxidant enzymes T-AOC and GSH. Whereas ERS is likely to be a major pathway responsible for DON-induced oxidative stress.ER is a multifunctional organelle that implements and participates in several essential biological functions, including protein synthesis, steroid hormone synthesis, post-translational protein modification, peptide chain folding, glucose concentration regulation, calcium homeostasis, as well as lipid metabolism [77]. When the ability of cells to correctly fold and post-translationally modify secretory and transmembrane proteins in the ER is disrupted, resulting in an accumulation of misfolded proteins in this organelle–a condition known as ERS- it is imperative that the ability of the cell to fold proteins to meet the demands of protein folding be restored rapidly in order for the cell to survive after ERS [78]. At this point, there is a quality control system at the ER that would eliminate unfolded or misfolded proteins from the secretory pathway and export the correctly folded proteins to their ultimate destinations exclusively, where the interrupted homeostasis within the ER involves a series of stress-responsive signalling pathways generically referred as UPR [79]. The UPR is composed of three pathways: inositol-requiring enzyme 1 (IRE1), activating transcription factor 6 (ATF6), and protein kinase R (PKR)-like endoplasmic reticulum kinase (PERK) [80]. The PERK is one of the four well-known kinases that respond to cellular stress by activating eIF2α, CHOP, or other signal transduction cascades [81]. After the separation of the PERK protein from GRP78 in the ERS process, PERK is activated by dimerisation and autophosphorylation, and its downstream eukaryotic translation initiation factor EIF2α is phosphorylated to form P-EIF2α [82]. At the same time, ERS leads to mitochondrial damage and increased ROS generation. It has been found that FB1 increased the levels of endoplasmic reticulum stress markers (Bip, ATF4 and CHOP) and activated the PERK-CHOP signalling pathway [83], 3-Ac-DON altered ultrastructural changes in the ER as well as enhanced protein levels of p-IRE1α, p-PERK, and downstream targets via mouse liver [24], which are analogous to our findings. While our works demonstrated that DON treatment increased the gene expression levels of GRP78, eIF2α, ATF4, and CHOP on the PERK/CHOP signalling pathway and increased the protein expression of PERK in cells, and the levels of these five proteins were declined noticeably by the combined treatment of DON + CUR, which suggests that CUR can alleviate DON-induced cellular ERS through the PERK/CHOP signalling pathway.

CUR has been used extensively in living organisms as a potent antioxidant and anti-inflammatory agent. The addition of CUR to broiler diets was found to increase the antioxidant capacity of meat, reduce lipid peroxidation and protein oxidation, and improve meat quality [84]. The addition of CUR to the diet of laying hens had anticoccidial effects and improved egg quality [85]. Dietary supplementation of 400 mg/kg CUR was also found to significantly increase total weight gain and total feed intake in IUGR piglets [86,87]; at the same time, feed-grade CUR is sold in powder form, and its local (China) market price is only about $8–11 per kg. According to different species (poultry, pigs, ruminants, etc.), the addition of CUR ranges from 150 to 800 mg/kg. For example, for pigs, the additional cost per ton can be limited to $3.2–4.4 at the rate of 400 mg/kg, which shows the great application potential of CUR in agricultural production. However, there are still some limitations that need to be addressed. The first is that CUR has a short lifespan in the animals. A single oral dose of 500 mg CUR per kg body weight in rats resulted in a maximum plasma CUR concentration of 0.06 µg/mL after 40 min, with an elimination half-life of 28 min [88]. The absorption half-life of CUR (2 g/kg) administered orally to rats was 0.31 ± 0.07 h, and the elimination half-life was 1.7 ± 0.5 h [89]. After a single oral dose of 10 or 12 g of CUR, only one person detected free CUR in the plasma 30 min after the dose [90]. The second is that CUR degrades rapidly and has a limited distribution. A large body of evidence has shown that the only organ exposed to high concentrations of CUR after oral administration is the gastrointestinal tract and that only small amounts of free CUR reach the peripheral blood [91]. In animal models of CUR, it has been suggested that few curcuminoids reach peripheral organs after oral administration [45]. Studies have shown that CUR is poorly absorbed by intestinal cells, and a small fraction is efficiently excreted via bile and kidney, while the majority is excreted in faeces [92]. In addition to CUR, of course, other widely used polyphenols that are structurally related to CUR and have good efficacy include quercetin, resveratrol and silymarin. Several studies have shown that growing pigs fed a diet supplemented with quercetin for 7 weeks showed an upward trend in feed conversion and an increase in total DNA methylation [93]. Dietary supplementation with resveratrol may be effective in improving meat quality in Peking ducks, in part because resveratrol stimulates intramuscular fat and flavour amino acid deposition and alters muscle fibre properties [94]. Silymarin supplementation in mycotoxin-contaminated broiler diets reduced end-of-production growth impairment, prevented oxidative stress, improved meat quality and increased polyunsaturated fatty acids [95]. These natural plant extracts showed significant improvements in animal performance and meat quality and are promising as potential feed additives.

## 4. Materials and Methods

### 4.1. Cell and Cell Culture

IPEC-J2 cells were obtained from the German Microbial Strain Preservation Center (DSMZ, Braunschweig, Germany). The cells were cultured in a complete medium consisting of 50 mL FBS and 5 mL double antibiotic (penicillin 10 KU/mL, streptomycin mg/mL) and added to an entire sealed bottle of DMEM/F12 medium. The ice-preserved IPEC-J2 cells were retrieved from the liquid nitrogen tank, resuspended in a complete DMEM/F12 medium and cultured in an incubator at 37 °C with 5% CO_2_. When cell integration reached 80%, the cells were transferred and stabilised for subsequent experiments.

### 4.2. Chemicals and Reagents

CUR (#C1386), DON (#D0156) and dimethyl sulfoxide (DMSO, #D2650) were obtained from Sigma-Aldrich (St. Louis, MO, USA). Fetal bovine serum (FBS, #PWL001), DMEM/F12 medium (#MA0214), penicillin/streptomycin (#MA0110) and cell counting kit-8 (CCK8, #MA0218) were procured from Meilun Biotechnology (Meilun, Dalian, China). The kits for MDA (#A003-4), T-AOC (#A025-2) and GSH (#A006-2) were purchased from Jiancheng (Jiancheng Bioengineering Institute, Nanjing, China). Reactive oxygen species assay kit (ROS, #S0033S), mitochondrial membrane potential assay kit with JC-1 (JC-1, #C2006), annexin V-FITC apoptosis detection kit (#C1062S) and propidium iodide (PI, #ST511) were obtained from Beyotime (Beyotime, Shanghai, China). The NF-κB p65 (#66535-1), IL-8 (#27095-1), IL-1β (#16806-1), IL-6 (#21865-1), TNF-α (#60291-1), Bax (#50599-2), Bcl-2 (#60178-1), Caspase-3 (#66470-2), Caspase-9 (#66169-1), β-Actin (#81115-1), ATF4 (#60035-1), PERK (#68482-1), CHOP (#66741-1), GRP78 (#66574-1), eIF2α (#11170-1), Goat Anti-mouse IgG (#SA00001-1) and Goat Anti-rabbit IgG (#SA00001-2) were acquired from Proteintech Group (Proteintech, Wuhan, China).

### 4.3. DON and CUR Treatment

CUR was dissolved in DMSO at a concentration of 60 μM for storage and diluted to 6, 8, 10, 12, 14, 16, 18 and 20 μM with cell medium for use. The same volume of DMSO was added to the control group, where the final concentration of DMSO is less than 0.1% in the treatment solution prepared above. DON was solubilised in DMSO at 10 mg/mL for storage and dissolved in cell medium for use at 0.1, 0.2, 0.4, 0.6, 0.8, 2.0 and 4.0 µg/mL. In subsequent experiments, the concentration of CUR was 14 μM, and the concentration of DON was 0.6 μg/mL to treat IPEC-J2 cells for 24 h.

### 4.4. Assessment of Cell Viability by CCK-8 Method

The viability of the cells was measured by using Cell Counting Kit-8. IPEC-J2 cells were plated in 96-well plates at a density of 1 × 10^6^ cells/well; when the density reached 70–80%, chemicals at different doses of DON and CUR were used to handle them for 24 h. Then 10 µL of CCK-8 was added to each well, which was incubated for 2 h at 37 °C. Viability was calculated by measuring the optical density (OD) at 450 nm for each well.

### 4.5. Measurement of Reactive Oxygen Species (ROS)

ROS detection was performed using the fluoroprobe DCFH-DA diluted in serum-free medium to a final concentration of 10 µmol/L. After 24 h of treatment with CUR (14 µM), DON (0.6 µg/mL) and CUR (14 µM) + DON (0.6 µg/mL), the medium was withdrawn and an appropriate dose of DCFH-DA working solution was added to the six-well plates. Plates were then incubated at 37 °C, 5% CO_2_ for 30 min under light protection with three washes of serum-free medium to adequately remove unincorporated DCFH-DA from the cells, immediately followed by fluorescence microscopy.

### 4.6. Determination of Oxidative Stress Indices

Glutathione (GSH) activity was determined by the colorimetric method using 5, 5′-dithiobis-p-nitrobenzoic acid. Malondialdehyde (MDA) levels were tested as an indicator of lipid peroxidation via a 2-thiobarbituric acid colour reaction. Total antioxidant capacity (T-AOC) was measured by the 2,2′-azino-bis (3-ethylbenzothiazoline-6-sulphonic acid) oxidation method. The indicators were tested strictly according to the kit instructions.

### 4.7. Mitochondrial Membrane Potential (MMP) Assay

IPEC-J2 cells with CUR and DON treatment for 24 h, the culture medium was discarded, the cells were washed once with PBS (1×), and to each well of the 6-well plate, 1 mL of cell culture fluid and 1 mL of JC-1 staining working buffer were added, thoroughly mixed, incubated at 37 °C for 20 min without light, after the completion of the incubation, the supernatant was aspirated, washed twice with JC-1 staining working buffer (1×), which was immediately observed with fluorescence microscope.

### 4.8. Total RNA Extraction and Quantitative Real-Time PCR (RT-qPCR)

The cells were plated in a 6-well plate at a density of 2 × 10^5^ cells per well. When the cell growth density reached 70–80%, the medium was discarded, the appropriate concentration of DON and CUR working solution was added to treat the cells for 24 h, the cell culture medium was discarded, washed twice with PBS, 500 μL of TRIZOL lysate was added, and the lysate was collected in the new centrifuge tube. Total RNA was extracted according to the Total RNA Extraction Kit and then reverse transcribed to cDNA using the Reverse Transcription Kit. The reaction conditions were as follows: 42 °C, 15 min, 95 °C, 3 min. The relative mRNA expression of related genes was detected by real-time quantitative fluorescence PCR. The reaction conditions were as follows: pre-denaturation at 95 °C, 15 min, annealing at 95 °C, 5 s at 60 °C, annealing at 60 °C, extension at 72 °C for 10 s, a total of 40 cycles. The sequence of the corresponding primers is shown in Table 1. The relative mRNA expression of the target gene was calculated using the 2^−ΔΔCt^ method.

### 4.9. Extraction of Total Cellular Protein and Western Blotting Analysis

The cells were inoculated into a 6-well plate at a density of 2 × 10^5^ cells per well. When the cell growth density reached 70–80%, the medium was discarded, the appropriate concentration of DON and CUR working solution was added to treat the cells for 24 h, the cell culture medium was discarded, and the cell culture medium was washed twice with cold PBS. After the appropriate amount of protein extract was added, the lysed 10 min was collected into a centrifuge tube, centrifuged at 16,000× *g* for 10 min, and the supernatant was retained. The protein concentration was then determined using the BCA method. Then denatured at 100 °C. The protein sample was subjected to non-blocking PAGE gel electrophoresis for 2 h, and the protein was transferred to the PVDF membrane by the semi-dry transfer method. The target protein band was cut out according to the molecular weight of the target protein and sealed with a protein-free rapid-sealing solution. The primary antibodies (NF-κB p65, IL-8, IL-1β, IL-6, TNF-α, Bax, Bcl-2, caspase-3, caspase-9, β-actin, ATF4, PERK, CHOP, GRP78 and eIF2α) were then refrigerated and incubated overnight. The secondary antibodies (goat anti-mouse IgG and goat anti-rabbit IgG) were incubated at room temperature after three TBST washes, and the PVDF membrane was soaked in Omni-ECL ultra-sensitive chemiluminescence solution after antibody incubation. The Tanon 4600 series automated chemiluminescence image analysis system was used for imaging, and Image J software was used for band analysis.

### 4.10. Apoptosis Detection Using Flow Cytometry

IPEC-J2 cells were conditioned with CUR and DON for 24 h and discarded the medium, which was digested with trypsin without EDTA. Cells were gently blown down with cell culture medium and transferred to a centrifuge tube, and 1000× *g* centrifugation 5 min was used to collect cells. The centrifuged cells were washed twice with a pre-cooled PBS solution. The cells were gently resuspended with the addition of 195 μL Annexin V-FITC binding solution. Add 5 μL Annexin V-FITC, mix gently, then add 10 μL propidium iodide staining solution, mix gently, incubate for 20 min at room temperature in the dark and detect by flow cytometry after staining.

### 4.11. Statistical Analysis

The data were analysed using SPSS (version 22, IBM Corporation, Armonk, NY, USA) software, with results expressed as means ± SEMs with at least three biological replicates, and the statistical significance of figures was tested by one-way analysis of variance (ANOVA) and charted using GraphPad PRISM software (version 8.0, GraphPad, Inc, San Diego, CA, USA), with *p* < 0.05 indicating statistical significance.

## 5. Conclusions

In conclusion, our studies suggest that CUR may suppress the activities of TNF-α, NF-κB, apoptosis and ERS by interfering with the cross-talk of its targets with DON-activated cytotoxic factors. The above results indicated that the mitigation of DON toxicity by CUR is a systematic inhibitory system consisting of multiple pathways through data exploration and experimental validation aiming to confirm the multifaceted nature of CUR’s antitoxic effects; these studies provide a preliminary experimental basis for the potential protective mechanism of CUR against DON-induced IPEC-J2 cell damage, suggesting that CUR could be used as a novel feed additive to protect livestock and poultry from the harmful effects of DON.

## Figures and Tables

**Table 1:** Sequences and Parameters of the Primers Used for RT-qPCR.

Gene Symbols	Nucleotide Sequence of Primers (5′→3′)	Product Length (bp)	Accession No.
β-Actin	F: CTGGAACGGTGAAGGTGA R: TTTGGAAAGGCAGGGACT	218	XM_021086047.1
IL-1β	F: CCACAAATCTCTAGTGCTGGCT R: CAGGGTGGGCGTGTTATCT	199	XM_021085847.1
NF-κB	F: AAGAGCAGCGTGGTGGGCAGTG R: CCGGAACGGTCTCCATCACAATC	163	XM_021079371.1
IL-6	F: AGGCCGTGCAGATTAGTACC R: ATTTGTGGTGGGGTTAGGGG	95	NM_001252429.1
IL-8	F: GCCTTCTTGGCAGTTTTCCTG R: TGGAAAGGTGTGGAATGCGTA	113	NM_213867.1
TNF-α	F: TTATCGGCCCCCAGAAGGAA R: CGACGGGCTTATCTGAGGTT	102	NM_214022.1
GRP78	F: ATATAAGCGGAGCAGGCGAC R: GAGCTCTCACACACACGGAA	87	XM_001927795.7
eIF2α	F: AGAATGCCGGGTCTGAGTTG R: GGATACGCCTTCTGGAGAGC	172	XM_001928339.4
ATF4	F: AGTCCTTTTCTGCGAGTGGG R: CTGCTGCCTCTAATACGCCA	80	XM_021090887.1
CHOP	F: AGCTCTGATTGACCGGATGG R: AAGGTCAGCAGTAGCCCAAG	83	XM_005674378.2

β-Actin was used as a housekeeping/control gene.

## Data Availability

The data that support the findings of this study are available from the corresponding author upon reasonable request.
